# Hand Grip Strength: age and gender stratified normative data in a population-based study

**DOI:** 10.1186/1756-0500-4-127

**Published:** 2011-04-14

**Authors:** Nicola M Massy-Westropp, Tiffany K Gill, Anne W Taylor, Richard W Bohannon, Catherine L Hill

**Affiliations:** 1 University of South Australia, School of Health Sciences Adelaide, South Australia SA 5000, Australia; 2 Population Research and Outcome Studies Unit, Department of Health, Adelaide, South Australia, Australia; 3 Department of Medicine, University of Adelaide, Adelaide, South Australia SA 5000, Australia; 4 Department of Kinesiology, University of Connecticut, Storrs, CT 06269, USA; 5 Rheumatology Unit, The Queen Elizabeth Hospital, 28 Woodville Rd, Woodville South Australia 5011, Australia; 6 The Health Observatory, University of Adelaide, Adelaide, South Australia SA 5000, Australia

## Abstract

**Background:**

The North West Adelaide Health Study is a representative longitudinal cohort study of people originally aged 18 years and over. The aim of this study was to describe normative data for hand grip strength in a community-based Australian population. Secondary aims were to investigate the relationship between body mass index (BMI) and hand grip strength, and to compare Australian data with international hand grip strength norms.

**Methods:**

The sample was randomly selected and recruited by telephone interview. Overall, 3 206 (81% of those recruited) participants returned to the clinic during the second stage (2004-2006) which specifically focused on the collection of information relating to musculoskeletal conditions.

**Results:**

Following the exclusion of 435 participants who had hand pain and/or arthritis, 1366 men and 1312 women participants provided hand grip strength measurement. The study population was relatively young, with 41.5% under 40 years; and their mean BMI was 28.1 kg/m^2 ^(SD 5.5). Higher hand grip strength was weakly related to higher BMI in adults under the age of 30 and over the age of 70, but inversely related to higher BMI between these ages. Australian norms from this sample had amongst the lowest of the hand grip strength of the internationally published norms, except those from underweight populations.

**Conclusions:**

This population demonstrated higher BMI and lower grip strength in younger participants than much of the international published, population data. A complete exploration of the relationship between BMI and hand grip strength was not fully explored as there were very few participants with BMI in the underweight range. The age and gender grip strength values are lower in younger adults than those reported in international literature.

## Background

Hand grip strength can be quantified by measuring the amount of static force that the hand can squeeze around a dynamometer. The force has most commonly been measured in kilograms and pounds, but also in millilitres of mercury and in Newtons.

Hand grip strength is a reliable measurement when standardised methods and calibrated equipment are used, even when there are different assessors [1] or different brands of dynamometers [2].There are different methods of positioning patients during measurement, and for calculating their grip strength from repeated measures, so the American Society for Surgery of the Hand and the American Society of Hand Therapists [3] have standardized positioning, instruction and calculation of grip strength.

Published normative data for hand grip strength are available from many countries, and in most cases, data are divided into age and gender subgroups [4-7]. Analysis of grip strength by gender shows higher grip by males at all ages, and analysis by age group demonstrates a peak of grip strength in the fourth decade and then a gradual decline in grip strength for both genders [4-7]. This trend is always present even though some studies divide participants by age gender, and then by right and left hand, while a small number of studies divide participants by age gender and then dominant and non-dominant hand (5).

Grip strength is related to and predictive of other health conditions, although the relationship is not stated to be causative [4,8]. Normal hand grip strength is positively related to normal bone mineral density in postmenopausal women, [9] with some researchers suggesting that grip strength be a screening tool for women at risk of osteoporosis [10]. Longitudinal studies suggest that poor grip strength is predictive of increased mortality from cardiovascular disease and from cancer in men, even when factors of muscle mass and body mass index are adjusted for [11,12]. Hand grip strength is negatively associated with physical frailty even when the effects of body mass index (BMI) and arm muscle circumference are removed [13]. Researchers have suggested that the factor related to frailty and disability in later life is the manner in which muscles are used, and this can be measured by hand dynamometry [13].

Disparity exists in the literature over the relationship between hand grip strength and BMI, many researchers claiming a positive relationship between grip strength and BMI in both genders and all ages, while other researchers found no relationship [14-17]. The studies were from different countries, and involved participants of different ages, genders, ethnicities, types of work and access to food. There is one study of hand grip strength in Australian adults [18], but no exploration of the relationship between grip strength and BMI in an Australian population.

The aim of this study was to describe normative data for hand grip strength of an Australian population. Secondary aims were to investigate the relationship between BMI and hand grip strength, and to compare Australian data with international hand grip strength norms.

## Method

Prior to the study commencing, approval for the research was obtained from the North West Adelaide Health Service Ethics of Human Research Committee and informed consent was obtained from each participant, conforming to the Helsinki Declaration.

Data were obtained from the North West Adelaide Health Study (NWAHS). In stage one (2000-2003), participants over the age of 18 years were randomly selected using the electronic white pages telephone directory, interviewed and invited to attend a clinic for physical assessment. In stage two (2004-2006), participants were re-contacted, invited to complete telephone interview, complete a self-administered questionnaire and have a clinical assessment. Overall, 3 206 (81% of those recruited) participants returned to the clinic during the second stage (2004-2006) which specifically focused on the collection of information relating to musculoskeletal conditions [19].

Participants were asked if they had ever had pain or aching in their shoulder, arm or hand at rest or when moving, on most days for at least a month and if they had ever had stiffness when getting out of bed in the morning on most days for at least a month as part of the Computer Assisted Telephone Interview. Participants who answered positively to either of these questions were removed from the grip strength analysis. BMI was calculated from height and weight measurements taken at the clinic assessment. Information relating to hand dominance (participants were asked "What is your dominant hand") was also collected as part of the clinic assessment.

### Measurement

Grip strength was measured using a Jamar Analogue Hand Dynamometer with participants seated, their elbow by their side and flexed to right angles, and a neutral wrist position, the dynamometer handle position II and provision of support underneath the dynamometer. This position, followed by calculation of the mean of three trials of grip strength for each hand, has been well-documented as reliable [3]. Five assessors were trained in the use of the dynamometer according this protocol and practiced the testing procedure prior to assessments. The participants' hand grip strength data were displayed as left or right regardless of hand dominance.

Participants' BMI was calculated following the measurement of each participant's weight and height.

### Instrument

Dynamometers were stored carefully in their custom made cases but if knocked, they were tested and recalibrated by biomedical engineers.

Published hand grip strength norms were sought through Google Scholar, EbscoHost and Medline, as well as searching reference lists of relevant papers. Key words in all combinations were 'hand grip strength, norm*, dynamometer'. Data were accepted for comparison if subjects were screened, measured and their values calculated in the same manner as in this study [3]. Data were not accepted if there was not a representation of many ages, if left and right hands were not presented separately, if different dynamometers were used or if subjects were injured or malnourished.

### Statistical Analysis

Participants were included in the analysis if they did not have hand pain, osteoarthritis or rheumatoid arthritis. They were stratified by gender and then by age into ten year subgroups from 20 years until the age of 70 years and over.

Analyses of grip strength were undertaken by age and gender and are presented by left hand or right hand. Mean and standard deviation of grip strength in kilograms were calculated as the range in kilograms for each group was normally distributed.

Body Mass Index scores and right hand grip strength for each age group and gender were compared by Pearson r correlation, with a significance level of 0.05.

## Results and Discussion

Overall, 3206 men and women aged 20 years and over participated in clinical assessment in Stage 2. Of these, 436 persons were excluded from this analysis due to the presence of arthritis and/or hand pain hand lasting over one month. Ninety-two respondents did not undertake a grip strength test, resulting in a total of 1314 men and 1315 women participants who provided a strength measurement. The participant group was relatively young, with 41.5% under 40 years; and the mean BMI was 28.1(5.5), with a range of 14.6 to 60.1. The standard deviations for all group means were small, therefore, using this data it is reasonable to predict a (pain-free) individual's grip strength if their age and gender are known.

Of the participants 89% were right-handed, 10% were left-handed and 1% did not state their hand dominance. It was not feasible to provide grip strength by dividing participants into gender groups, age groups and then into left and right-handed groups, as the number of left handed participants was only 270 in total. Thus the values for left hand and right hand grip strength, regardless of hand dominance, are presented in Table 1.

**Table 1:** Mean and Standard Deviation and Hand Grip Strength in kilograms, for men and women, presented in ascending age groups

Men				Women			
**Age**	**right**	**left**	**BMI**	**Age**	**right**	**left**	**BMI**

20 to 29	47(9.5)	45(8.8)	26.4(5.1)	20 to 29	30(7)	28(6.1)	25.1(5.8)
30 to 39	47(9.7)	47(9.8)	28.3(5.2)	30 to 39	31(6.4)	29(6)	27.3(6.8)
40 to 49	47(9.5)	45(9.3)	28.4(4.6)	40 to 49	29(5.7)	28(5.7)	27.7(7.7)
50 to 59	45(8.4)	43(8.3)	28.7(4.3)	50 to 59	28(6.3)	26(5.7)	29.1(6.4)
60 to 69	40(8.3)	38(8)	28.6(4.4)	60 to 69	24(5.3)	23(5)	28.1(5.1)
70 +	33(7.8)	32(7.5)	27.2(3.9)	70 +	20(5.8)	19(5.5)	27(4.7)

A very weak positive relationship was found between higher BMI and right hand grip strength the youngest and oldest adults in the sample. For young adults and those in their fourth, fifth and sixth decade, a higher BMI was inversely related to hand grip strength, (Table 1).

**Figure 1:**
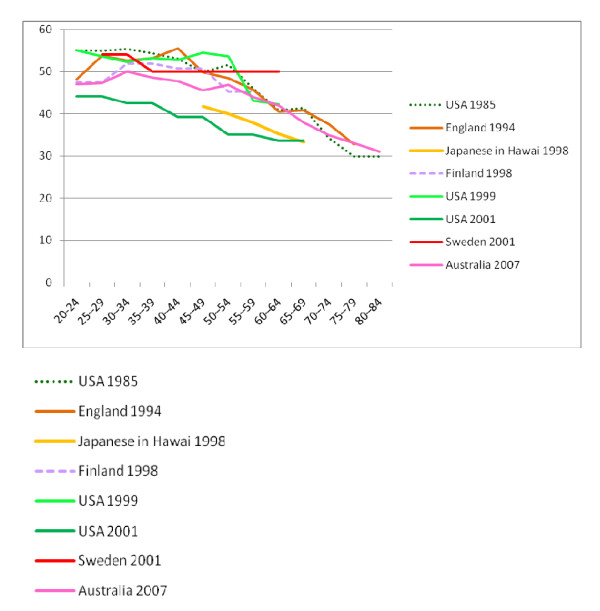
**Comparison of NWAHS and International Right Hand Grip Norms for Men**.

**Figure 2:**
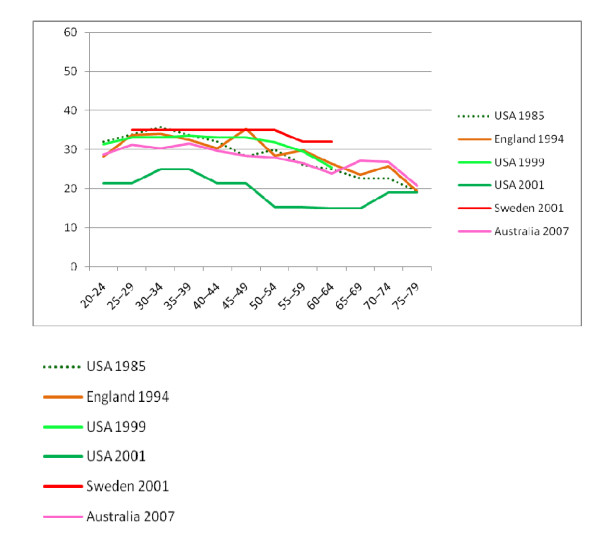
**Comparison of NWAHS and International Right Hand Grip Norms for Women**.

Seven published studies [7,20-25] were accepted for comparison with the current data and eighteen studies were excluded as they used different equipment, measurement position, or they did not divide subjects by age or by which hand was measured. There are considerable differences between the grip data (Figures 1 and 2), even though all participants were screened to exclude those with upper limb conditions. In addition, no studies were included if participants had chronic illness or malnutrition. Possible reasons for the differences are in the recruitment locations; for example the lowest *and *highest results were obtained from the USA, but the strongest were recruited in public places [7] and the lowest were recruited from doctor's offices [17].

Past research exploring the relationship between BMI and hand grip strength has provided incongruent findings. Published data suggests that higher BMI in adults under 25 and over 70 years is positively correlated with higher grip strength, and being overweight (BMI over 25) and obese ranges (BMI over 30), in adults between the age of 25 and 70 is correlated with lower hand grip strength. These trends can be seen in the current study also, but there are no moderate or large correlations. The current study cannot fully investigate the relationship of BMI and strength, as only 27 participants exhibited low BMI. Conversely, published studies set in India [15,16] Africa [17] and Japan [20] could not fully explore the relationship between BMI and hand grip strength because none of the subjects' BMI exceeded 25.

The relationship between BMI and low hand grip strength is further explored in a study that divided participants with low BMI (≤18.5) into two health status groups of 'chronically undernourished' and 'underweight' [15]. The chronically undernourished groups have significantly lower hand grip strength than the underweight groups, both being significantly less strong than the 'well nourished' groups of BMI higher than 18.5. Analysis of this subgroup was not feasible in the current study as there were only four men and 27 women with a BMI under 18.5.

## Conclusion

This study provides a large sample of normative data for clinical use in hand and upper limb rehabilitation, and possible screening for other health issues. It explores the relationship of grip strength with elevated BMI and found no significant relationships. The study compares the Australian sample with international grip strength norms, finding these population-based norms to be lower than international convenience samples.

## Competing interests

The authors declare that they have no competing interests.

## Authors' contributions

NMW formatted the data, and completed the database search for other normative data, made data comparisons with published data and suggested that the grip strength data be made available through publication, all authors agreed.

TG, AT and CH applied for the grant for this study, designed the study methods then TG handled and organized the large data files generated from the study. TG and NMW independently carried out the statistical analyses.

TG, RB and CH independently evaluated the statistical analyses made by NMW and wrote commentary on the manuscript.
